# Body Composition Differences Among Individual, Team, and Combat Sport Athletes Enrolled in a Spanish Sports Technification Program

**DOI:** 10.3390/jfmk11030299

**Published:** 2026-07-29

**Authors:** Jorge Alarcón-Jimenez, Alberto Pardo-Ibáñez, Nieves de Bernardo, Laura García-Martínez, Javier Gámez-Payá, Javier Bonastre-Férez, Benjamín Cuenca, José Miguel Soriano, Carlos Villarón-Casales

**Affiliations:** 1 Department of Physiotherapy, Universidad Católica de Valencia, 46900 Valencia, Spain; nieves.debernardo@ucv.es (N.d.B.); laura.gmartinez@ucv.es (L.G.-M.); benjamin.cuenca@ucv.es (B.C.); 2 Department of Physical Education and Sport, University of Valencia, 46000 Valencia, Spain; alberto.pardo@uv.es; 3 Department of Physiotherapy, Nutrition and Sports Sciences, Faculty of Health Sciences, Universidad Europea de Valencia, 46010 Valencia, Spain; javier.gamez@universidadeuropea.es (J.G.-P.); javier.bonastre@universidadeuropea.es (J.B.-F.); carlosalberto.villaron@universidadeuropea.es (C.V.-C.); 4 Joint Research Unit in Endocrinology, Nutrition and Clinical Dietetics, University of Valencia—Health Research Institute La Fe, 46026 Valencia, Spain; jose.soriano@uv.es; 5 Evidence and Experimentation-Based Food Science Group (CiAlBEx), Institute of Materials Sciences, Scientific Park, University of Valencia, 46026 Paterna, Spain

**Keywords:** body composition, anthropometry, youth athletes, sports performance, team sports, combat sports, swimming, triathlon

## Abstract

**Background:** Sports training induces functional and structural adaptations that may influence body composition, particularly during adolescence when growth and maturation are still occurring. Although anthropometric characteristics have been extensively studied within individual sports, comparative analyses among different sport categories remain limited. The aim of this study was to compare body composition characteristics among athletes participating in individual, team, and combat sports within a Spanish Sports Technification Program. **Methods:** A total of 118 young athletes (62 males and 56 females) from the Valencian Sports Technification Centre participated in this cross-sectional study. Participants were classified into three sport categories: individual sports (athletics, swimming, and triathlon), team sports (handball and volleyball), and combat sports (karate and taekwondo). Anthropometric assessment was performed according to the International Society for the Advancement of Kinanthropometry (ISAK) protocol. Body composition variables, including fat mass, muscle mass, and bone mass, were estimated using established anthropometric equations. Statistical analyses were performed using non-parametric tests, with significance set at *p* < 0.05. **Results:** After adjustment for multiple comparisons, significant differences remained between sport categories for several body composition indicators. Athletes from the individual sport group showed a higher muscle mass percentage and lower body fat percentages estimated using the Withers, Faulkner, and Carter equations than those from the team sport group. Similarly, estimated body fat percentages were lower in the individual sport group than in the combat sport group across all three equations. Team sport athletes presented higher absolute fat and muscle mass than combat sport athletes. However, some differences identified in the initial analyses, including fat mass and bone mass percentage between the individual and team sport groups and muscle mass percentage between the team and combat sport groups, did not remain statistically significant after adjustment for multiple comparisons. **Conclusions:** Body composition characteristics differed among the groups of young athletes participating in the specific individual, team, and combat sports included in this study. Within this cohort, athletes from the individual sport group generally exhibited lower body fat levels than those from the team and combat sport groups, whereas differences were also observed in other anthropometric characteristics. These findings should be interpreted within the context of the specific sports included and should not be generalized to individual, team, or combat sports as a whole. The findings may assist coaches, sports scientists, and health professionals in the monitoring and development of young athletes participating in similar sports and development programs.

## 1. Introduction

Sport has gained a prominent position in modern society, largely due to the social recognition achieved by athletes, a phenomenon that has been reinforced by the media and has contributed to the increasing popularity of sport. This growing interest has led to greater specialization from an early age and has promoted the development of training programs for talented young athletes [1].

Numerous studies have focused on the anthropometric and physiological characteristics of athletes, demonstrating that potential sporting talent can be identified on the basis of these characteristics, while athletic performance may subsequently be enhanced through training and technical development [2,3,4,5].

Body composition analysis provides valuable information about the relative proportions of the main body components. It allows the assessment of changes associated with age, growth, physiological and pathological conditions, as well as adaptations related to sports participation.

Several studies have highlighted the difficulty of distinguishing between changes resulting from normal growth and maturation processes—such as the increase in fat mass observed in girls or the pubertal increase in muscle mass in boys—and those induced by sports training [6].

Body composition is a relevant variable in athlete monitoring and is associated with sport-specific performance demands [7,8,9,10]. The distribution of fat mass, muscle mass, and bone tissue influences physical capacities such as strength, endurance, power production, and movement efficiency, while also providing relevant information for training prescription and nutritional monitoring [7,8,9,10]. Consequently, the regular assessment of body composition is widely recommended throughout the athlete development process [7,8,9,10].

The interpretation of these variables is particularly challenging during adolescence, a period characterized by rapid physiological changes associated with growth and biological maturation. Increases in muscle mass, changes in fat distribution, and skeletal development occur simultaneously with sport-specific training adaptations, making it difficult to establish the relative contribution of maturation and exercise to the observed anthropometric profile [11]. For this reason, standardized assessment procedures are necessary to allow meaningful comparisons between athletes and over time [11].

Anthropometric evaluation following the recommendations of the International Society for the Advancement of Kinanthropometry (ISAK) offers a practical, non-invasive, and cost-effective method for estimating body composition in both clinical and sporting environments [12]. The use of standardized measurement protocols reduces technical variability and facilitates the generation of reliable reference values that can be applied to talent identification programs, longitudinal athlete monitoring, and individualized performance optimization [7,13].

Although numerous studies have reported anthropometric characteristics in isolated sporting disciplines, considerably less attention has been devoted to comparisons across broader sport categories that differ substantially in their physiological and biomechanical demands [7,8,9,10,14]. Establishing body composition profiles for athletes engaged in individual, team, and combat sports may therefore improve the interpretation of anthropometric data and provide practitioners with discipline-specific reference values that support evidence-based decision-making in youth sports [7,8,9,10,14].

Given the composition of the available cohort, these comparisons were intended as exploratory analyses of the specific sets of sports represented within each predefined category, rather than as an attempt to establish body composition profiles representative of individual, team, or combat sports as a whole.

Although body composition has been extensively described in individual sporting disciplines, direct comparisons between athletes from different sport categories assessed under identical methodological conditions remain limited. The present study contributes by evaluating athletes enrolled in the same sports technification program, assessed by the same accredited anthropometrist using standardized ISAK procedures, thereby reducing methodological variability that commonly affects comparisons across independent studies.

The aim of the present study was to describe and compare body composition indicators among athletes participating in individual sports (athletics, swimming, and triathlon), team sports (handball and volleyball), and combat sports (karate and taekwondo), according to Pino’s proposal [15], within the Sports Technification Centre of Cheste (Spain). Although anthropometric characteristics have been widely investigated within specific sports disciplines, comparative analyses across broader sport categories such as individual, team, and combat sports remain scarce, particularly in adolescent athletes enrolled in sports technification programs. The contribution of the present study lies not merely in demonstrating that body composition differs among athletes from different sports, but in providing a direct comparison of these sport categories within a single cohort of selected young athletes assessed under the same methodological conditions. All participants belonged to the same structured sports technification program and were evaluated using standardized anthropometric procedures, thereby reducing methodological variability associated with comparisons across independent studies, populations, and assessment protocols. Furthermore, the simultaneous evaluation of multiple body composition components and the use of three different anthropometric equations for estimating body fat provide complementary information regarding the consistency of the observed differences across assessment approaches. Establishing comparative body composition data in this population may contribute to a more contextualized interpretation of anthropometric characteristics and support individualized decisions regarding training prescription and athlete development.

## 2. Materials and Methods

### 2.1. Participants

A total of 118 young athletes participated in this cross-sectional study, including 62 males and 56 females. The study was designed to compare body composition characteristics among athletes participating in different sport categories within the same Sports Technification Program.

The Valencian Sports Technification Centre of Cheste forms part of the Spanish Sports Technification Program, a public initiative promoted by the regional sports authorities with the aim of supporting the development of talented young athletes. Admission to the program is restricted to athletes who have demonstrated a high competitive level in their respective disciplines and who fulfill the selection criteria established by the corresponding regional and national sports federations. In addition to receiving specialized sport-specific training, athletes combine their academic education with structured high-performance training under the supervision of qualified technical staff.

Athletes trained from Monday to Friday and completed two daily training sessions on two days per week. According to the classification proposed by Pino [15], participants were grouped into three sport categories: individual sports, including athletics, swimming, and triathlon; team sports, including handball and volleyball; and combat sports, including karate and taekwondo.

The seven sports included in the study corresponded to the disciplines represented in the available cohort of athletes enrolled in the Sports Technification Program and were therefore not purposively selected to represent the full spectrum of individual, team, or combat sports. Accordingly, the three-category classification was used as an a priori analytical framework, and category-level comparisons should be interpreted within the specific composition of the present cohort.

Before the study was conducted, permission to carry out the research was obtained from the General Directorate of Sport of the Generalitat Valenciana, which was responsible for the Sports Technification Program and the participating athletes. According to the institutional requirements and applicable regulations at the time the study was conducted, formal review and approval by an institutional ethics committee were not required for this study. Therefore, no ethics committee approval code or approval date is available. All procedures were conducted in accordance with the ethical standards applicable to research involving human participants. Written informed consent was obtained from the parents or legal guardians of all participants. In addition, the study procedures were explained verbally to the athletes using age-appropriate language to ensure their understanding of the study and any potential discomfort associated with participation.

The classification into individual, team, and combat sports was established a priori as the primary grouping framework of the study, in accordance with the classification proposed by Pino [15]. The specific disciplines included in each category were determined by the sports represented among the athletes enrolled in the Sports Technification Program and available for inclusion in the study, rather than being purposively selected as representative of the full spectrum of sports within each category. Therefore, the included disciplines should not be considered representative of all individual, team, or combat sports. The purpose of this grouping was to compare body composition characteristics across broad sport categories rather than to establish homogeneous anthropometric profiles for each individual discipline. Nevertheless, we acknowledge that substantial within-category variability may exist. In particular, athletes participating in team sports may differ in their anthropometric characteristics according to their specific playing positions, while athletes participating in combat sports may differ according to competitive weight category. Information on playing position and weight category was not available for the present analysis, and participants could therefore not be further stratified according to these sport-specific characteristics. Consequently, the comparisons presented in this study should be interpreted as reflecting differences between broad sport categories, while acknowledging the potential heterogeneity of the individual disciplines and athletes included within each category.

### 2.2. Anthropometric Assessment

Anthropometric measurements were performed according to the protocol established by the International Society for the Advancement of Kinanthropometry (ISAK) [12]. The use of this standardized protocol ensured consistency in the measurement procedures and enhanced the comparability of anthropometric data across all participants and sport categories. All assessments were conducted by a Level 1 ISAK-accredited anthropometrist on the right side of the body, regardless of handedness or stance.

Each measurement was taken twice. The variation between repeated measurements was less than 1% for body mass, stature, girths, and breadths, and less than 5% for skinfold thicknesses. Participants were assessed barefoot and wearing light clothing.

The anthropometric variables included stature, measured using a stadiometer (SECA 225, Birmingham, UK); body mass, measured to the nearest 0.01 kg using a digital scale (SECA 770, Birmingham, UK); skinfold thicknesses (triceps, subscapular, biceps, iliac crest, supraspinale, abdominal, front thigh, and medial calf), measured using calibrated Harpenden calipers (John Bull, British Indicators, West Sussex, UK); girths (flexed and tensed arm, waist, hip, and calf), measured using an anthropometric tape (Lufkin W606PM, Cooper Hand Tools, Tyne & Wear, UK); and bone breadths (humerus and femur), measured using a Holtain anthropometer (Holtain Ltd., Dyfed, UK).

Body composition components were estimated using the equations proposed by Faulkner, Carter, and Withers. The technical error of measurement was calculated for all anthropometric variables and was lower than 3%, which is considered acceptable for anthropometric assessment [16].

The Faulkner, Carter, and Withers equations were selected because they are among the most widely used anthropometric models for estimating body fat percentage in athletic populations and have been extensively applied in studies involving adolescents and young athletes. Employing multiple predictive equations allowed the consistency of body fat comparisons across sport categories to be examined while facilitating comparisons with previously published studies. Although these equations do not constitute criterion methods for body composition assessment, they remain practical and valid tools for field-based evaluations when standardized ISAK procedures are followed.

### 2.3. Statistical Analysis

Statistical analyses were performed using SPSS Statistics version 19.0 (IBM Corp., Armonk, NY, USA).

The Kolmogorov–Smirnov test revealed that some variables did not meet the assumption of normality; therefore, non-parametric statistical procedures were applied. The primary statistical analyses were conducted on the combined sample of male and female athletes within each predefined sport category. Sex-specific inferential analyses were not performed because the limited sample sizes within several sport- and sex-specific subgroups would have resulted in insufficient statistical power and potentially unstable estimates. Consequently, the primary analyses should be interpreted as exploratory comparisons of the overall samples within each predefined sport category rather than as sex-specific comparisons. Detailed descriptive data stratified by sport discipline and sex are provided in Supplementary Tables S1–S3. Differences among sport categories were assessed using the Kruskal–Wallis test. When significant differences were identified, pairwise comparisons were performed using the Mann–Whitney U test. To account for the three pairwise comparisons conducted for each outcome, Bonferroni correction was applied to the corresponding *p*-values. Adjusted *p*-values below 0.05 were considered statistically significant. Effect sizes (r) were calculated from the standardized Z statistic for pairwise comparisons to facilitate interpretation of the magnitude of the observed differences. Approximate 95% confidence intervals for effect sizes were calculated using Fisher’s z transformation based on the corresponding effect size and the total sample size included in each pairwise comparison. The 95% confidence intervals were not adjusted for multiple comparisons and should therefore be interpreted as pointwise confidence intervals.

As an exploratory sensitivity analysis, body-size-adjusted anthropometric and body composition variables were additionally examined in the subset of athletes for whom complete individual ISAK assessment files were available (handball, volleyball, karate, and taekwondo). Anthropometric dimensions were standardized using ISAK Phantom Z-scores, while the fat mass index (FMI) and fat-free mass index (FFMI) were calculated by normalizing fat mass and fat-free mass to stature squared (kg/m^2^). Differences among the four sport disciplines were assessed using the Kruskal–Wallis test. When appropriate, pairwise comparisons were performed using the Mann–Whitney U test with Bonferroni adjustment. Because complete individual assessment files were not available for athletics, swimming, and triathlon, these analyses were considered exploratory and were not intended to reproduce the primary comparisons among individual, team, and combat sport categories.

## 3. Results

The demographic and anthropometric characteristics of the study participants are presented in Table 1. The descriptive body composition characteristics of athletes participating in individual, team, and combat sports are presented in Table 2, together with the statistical results of the comparisons between sport categories. More detailed descriptive data stratified by specific sport discipline and sex are provided in Supplementary Tables S1–S3. Overall, differences among sport categories were assessed using the Kruskal–Wallis test, followed, when appropriate, by pairwise comparisons using the Mann–Whitney U test. Bonferroni correction was applied to account for the three possible pairwise comparisons for each outcome, and adjusted *p*-values are reported in Table 2 and throughout the following sections.

The descriptive body composition characteristics of athletes participating in individual, team, and combat sports, together with the corresponding pairwise comparisons, are presented in Table 2. More detailed descriptive data stratified by specific sport discipline and sex are provided in Supplementary Tables S1–S3. Overall, the analyses revealed differences among sport categories for several body composition indicators. Initial comparisons were performed using the Kruskal–Wallis test, followed by pairwise analyses with the Mann–Whitney U test when appropriate. Bonferroni correction was applied to account for the three pairwise comparisons conducted for each outcome, and adjusted *p*-values are reported in Table 2 and throughout the following sections.

### 3.1. Comparison Between Individual and Team Sports

After adjustment for multiple comparisons, no significant differences were observed between the individual and team sport groups in fat mass expressed in kilograms (Z = −2.35, adjusted *p* = 0.056, r = −0.26 approximate 95% CI: −0.45 to −0.04) or bone mass percentage (Z = −2.18, adjusted *p* = 0.088, r = −0.25, approximate 95% CI: −0.45 to −0.03). However, athletes participating in the individual sport group showed a significantly higher percentage of muscle mass (Z = −2.67, adjusted *p* = 0.023, r = −0.30, approximate 95% CI: −0.49 to −0.08).

Significant differences were also observed in body fat percentage estimated using the Withers (Z = −3.91, adjusted *p* < 0.001, r = −0.44, approximate 95% CI: −0.60 to −0.24), Faulkner (Z = −3.06, adjusted *p* = 0.007, r = −0.34, approximate 95% CI: −0.52 to −0.13), and Carter (Z = −3.18, adjusted *p* = 0.004, r = −0.36, approximate 95% CI: −0.54 to −0.15) equations, with athletes from the individual sport group presenting lower estimated body fat values than those from the team sport group.

### 3.2. Comparison Between Individual and Combat Sports

No significant differences were identified between the individual and combat sport groups with respect to fat mass, bone mass, muscle mass, or their corresponding percentages.

However, body fat percentage estimated using the Withers (Z = −3.08, adjusted *p* = 0.006, r = −0.43, approximate 95% CI: −0.60 to −0.23), Faulkner (Z = −2.49, adjusted *p* = 0.038, r = −0.28, approximate 95% CI: −0.47 to −0.06), and Carter (Z = −2.63, adjusted *p* = 0.026, r = −0.30, approximate 95% CI: −0.49 to −0.08) equations was significantly higher in the combat sport group than in the individual sport group.

### 3.3. Comparison Between Team and Combat Sports

After adjustment for multiple comparisons, fat mass expressed in kilograms remained significantly higher in the team sport group than in the combat sport group (Z = −3.20, adjusted *p* = 0.004, r = −0.36, approximate 95% CI: −0.54 to −0.15; Mdn_team = 18.66 kg, Mdn_combat = 15.97 kg).

The difference in muscle mass percentage did not remain statistically significant after adjustment for multiple comparisons (Z = −2.09, adjusted *p* = 0.110, r = −0.23, approximate 95% CI: −0.43 to −0.01). However, muscle mass expressed in kilograms remained significantly higher in the team sport group (Z = −3.06, adjusted *p* = 0.007, r = −0.34, approximate 95% CI: −0.52 to −0.13; Mdn_team = 20.65 kg, Mdn_combat = 18.54 kg).

No significant differences were observed between the team and combat sport groups in body fat percentage estimated using the Withers, Faulkner, or Carter equations.

### 3.4. Exploratory Body-Size-Adjusted Analysis

To further explore whether differences in anthropometric characteristics could be influenced by overall body size, an exploratory sensitivity analysis was performed using the available individual ISAK assessment files from handball, volleyball, karate, and taekwondo athletes. Of the 82 files initially available, two contained insufficient information for the calculation of all body-size-adjusted variables and were therefore excluded, resulting in a final sample of 80 athletes (17 handball, 24 volleyball, 20 karate, and 19 taekwondo athletes).

After standardization using ISAK Phantom Z-scores, most anthropometric variables did not differ significantly among the four sport disciplines. Significant overall differences were identified only for medial calf skinfold Phantom Z-score (*p* = 0.013), calf girth Phantom Z-score (*p* = 0.022), and humerus breadth Phantom Z-score (*p* = 0.026). Similarly, no statistically significant differences were observed for the body-size-adjusted body composition indices, including fat mass index (FMI) and fat-free mass index (FFMI). Detailed results of the exploratory body-size-adjusted analyses are presented in Supplementary Table S4.

These findings suggest that, within the subset of team and combat sport athletes included in this supplementary analysis, most anthropometric differences were attenuated after accounting for body size. However, because individual ISAK assessment files were not available for athletes participating in athletics, swimming, and triathlon, these analyses should be interpreted as exploratory and should not be considered a direct size-adjusted replication of the primary comparisons among individual, team, and combat sport categories.

## 4. Discussion

The present study compared body composition characteristics among young athletes participating in individual, team, and combat sports within a Spanish Sports Technification Program. Overall, the findings indicate differences in selected body composition indicators among the specific sport groups included in the study, with athletes from the individual sport group generally exhibiting lower estimated adiposity than those from the team and combat sport groups. These observations reinforce the concept that the morphological characteristics of young athletes are influenced not only by growth and biological maturation but also by the specific physiological and biomechanical demands associated with each sporting discipline [1,2,4,5].

The principal contribution of the present study lies not simply in identifying differences in body composition between athletes from different sports, but in providing direct comparisons across predefined sport categories within a single, well-characterized cohort assessed under identical methodological conditions. Unlike many previous investigations, which have reported anthropometric data from individual sports or compared results across independent cohorts, all athletes in the present study belonged to the same sports technification program, were evaluated during the same period, and were assessed by the same ISAK-accredited anthropometrist using standardized procedures. This approach reduces methodological variability and allows more consistent comparisons across the included sport categories, thereby providing contextual reference data that may assist future research and athlete monitoring in similar high-performance youth settings.

Although adolescence is characterized by substantial changes in body composition related to normal development, systematic exposure to different training stimuli may contribute to the emergence of sport-specific anthropometric profiles. Therefore, interpreting body composition data requires consideration of both the developmental stage of the athlete and the particular requirements of the discipline practiced. From this perspective, the present findings provide additional evidence supporting the use of standardized anthropometric assessment as a practical tool for monitoring young athletes enrolled in sports technification programs [1,2,9].

Within the individual sports category, triathletes demonstrated body composition values that were more similar to those of swimmers than to middle-distance athletes. This pattern is consistent with previous investigations comparing these populations and suggests that prolonged endurance-oriented training may promote comparable anthropometric adaptations despite differences in competitive demands and movement patterns [17,18,19]. The similarity between swimmers and triathletes may reflect the high aerobic component shared by both disciplines, characterized by sustained exercise volumes and substantial energy expenditure over extended training sessions.

Swimming has traditionally been associated with body fat percentages that are slightly higher than those reported for land-based endurance sports, a characteristic that has often been attributed to the specific environmental and biomechanical demands of aquatic performance [6,20]. In the present study, swimmers presented body fat percentages of 17.11 ± 2.78% in females and 14.35 ± 1.10% in males according to the Withers equation, values that were broadly consistent with those previously described in the literature. Comparable observations were obtained using the Faulkner and Carter equations, although direct comparisons between studies should be interpreted cautiously because different predictive models may produce different absolute estimates of body fat percentage. Nevertheless, the consistency across the three equations employed in the present investigation strengthens the interpretation of the observed trends.

By contrast, middle-distance athletes displayed lower adiposity values than swimmers, particularly when body fat was estimated using the Faulkner and Carter equations. These findings agree with previous reports indicating that reduced body fat is advantageous for endurance running because excessive adiposity may increase energy expenditure and impair movement economy during prolonged locomotion [21]. Consequently, the lower fat percentages identified in these athletes are compatible with the physiological requirements of middle-distance events and support the concept that endurance-based land sports favor leaner body composition profiles.

It is noteworthy that the individual sports category included disciplines with distinct technical and metabolic characteristics despite their common aerobic component. Therefore, the differences identified between swimmers, triathletes, and middle-distance athletes emphasize that body composition should not be interpreted solely according to broad sport classifications but should also consider the specific training characteristics and performance demands of each discipline. This observation may be particularly relevant when establishing reference values for talent identification or longitudinal athlete monitoring.

The lower estimated body fat percentages observed in the individual sport group compared with the team sport group remained statistically significant across the Withers, Faulkner, and Carter equations after adjustment for multiple comparisons. In contrast, the differences initially observed in absolute fat mass and bone mass percentage did not remain statistically significant after correction, highlighting the importance of distinguishing robust between-group differences from findings that may be sensitive to multiple testing. The differences in estimated adiposity may reflect, at least in part, the distinct physical and tactical demands of the specific sports represented in these groups. Unlike the endurance-oriented individual sports included in the present study, handball and volleyball require repeated high-intensity efforts combined with strength, power, agility, and technical performance. Nevertheless, considerable heterogeneity in body composition should be expected both between and within team sports, highlighting the importance of interpreting anthropometric assessments according to the specific sporting discipline and, where relevant, playing position [4,6,22].

The comparison between individual and combat sports also yielded noteworthy findings. Although no significant differences were identified in fat mass, bone mass, or muscle mass expressed either in kilograms or percentages, athletes participating in combat sports exhibited significantly higher body fat percentages when estimated using the Withers, Faulkner, and Carter equations. These observations suggest that the assessment of adiposity may vary depending on the predictive equation employed and underline the importance of interpreting body composition using complementary anthropometric approaches.

Combat sports represent a particular competitive context in which athletes must achieve an appropriate balance between body composition and sport-specific performance. Although weight management constitutes an important component of preparation for many combat disciplines, maintaining muscle mass and neuromuscular performance is equally essential for technical execution and power production [5]. Consequently, the higher body fat percentages identified in the present cohort should not necessarily be interpreted as unfavorable but rather as reflecting the distinct physiological and competitive demands associated with these sports.

Interestingly, no significant differences in body fat percentage were identified between the team and combat sport groups, whereas differences in absolute fat mass and muscle mass remained statistically significant after adjustment for multiple comparisons. By contrast, the initially observed difference in muscle mass percentage did not remain statistically significant after correction. This pattern suggests that athletes from these sport categories may display comparable relative adiposity while differing in absolute body composition measures, potentially reflecting differences in overall body size and tissue distribution. Nevertheless, given the heterogeneity of the specific disciplines included within each category, these findings should be interpreted cautiously and should not be generalized to team or combat sports as a whole. Overall, these observations reinforce the importance of evaluating body composition through multiple complementary variables rather than relying exclusively on body fat percentage.

An exploratory sensitivity analysis was additionally performed using body-size-adjusted anthropometric variables (ISAK Phantom Z-scores) together with fat mass index (FMI) and fat-free mass index (FFMI) in the subset of athletes for whom individual ISAK assessment files were available. After accounting for body size, most adjusted anthropometric variables, including FMI and FFMI, did not differ significantly among the analyzed sport disciplines. Significant differences remained only for the medial calf skinfold, calf girth, and humerus breadth Phantom Z-scores. These findings suggest that, within the analyzed team and combat sport disciplines, only a limited number of proportional anthropometric characteristics differed after adjustment for body size, whereas most adjusted body composition variables were comparable. However, because individual ISAK assessment files were unavailable for the athletics, swimming, and triathlon groups, these supplementary analyses should be interpreted as exploratory and should not be considered a direct size-adjusted replication of the primary comparisons among the predefined individual, team, and combat sport categories.

The absence of statistically significant differences in several body composition variables should be interpreted cautiously, as body composition during adolescence is influenced by multiple interacting factors, including biological maturation, sex, and sport-specific training. Because sex and biological maturation were not incorporated as covariates or stratification factors in the primary analyses, their potential contribution to the observed between-group variability cannot be excluded [1,2,6]. In addition, the relatively limited sample size within some sport-specific subgroups may have reduced the statistical power to detect smaller differences between categories. Consequently, confirmation of these findings in larger multicenter studies would be desirable.

An additional methodological aspect worth highlighting is the use of three different anthropometric equations to estimate body fat percentage. Although the Withers, Faulkner, and Carter equations yielded different absolute values, the direction of the observed differences was consistent across methods, with athletes from the individual sport group presenting lower estimated adiposity than those from the team and combat sport groups. Notably, these between-group differences remained statistically significant across all three equations after adjustment for multiple comparisons. This consistency across estimation approaches supports the interpretation of the observed adiposity patterns, although it does not eliminate the methodological limitations inherent to anthropometric prediction equations. Comparisons between studies should therefore be interpreted cautiously when different anthropometric equations or assessment protocols have been applied [1,12,16].

From an applied perspective, the body composition data reported in this study may provide contextual information for coaches, sports scientists, nutritionists, physiotherapists, and physicians involved in the monitoring of young athletes participating in similar sports and development programs. Rather than being used as prescriptive targets or rigid normative thresholds, these data may serve as comparative benchmarks to contextualize an athlete’s body composition profile within the specific sporting environment represented in this cohort. In practice, repeated anthropometric assessments may help practitioners identify individual changes in fat and muscle mass over time, evaluate whether these changes are consistent with the athlete’s training demands and developmental trajectory, and identify unexpected variations that may warrant further assessment of training load, nutritional intake, or recovery strategies. Importantly, decisions regarding training or nutrition should not be based on isolated comparisons with group averages but should prioritize within-athlete longitudinal changes and consider sex, biological maturation, sport-specific demands, playing position, and competitive weight category when relevant. Thus, the main practical value of the present findings lies in supporting the contextual interpretation of standardized anthropometric monitoring rather than defining optimal body composition targets for young athletes [1,2,6,12].

Future studies should adopt longitudinal designs and incorporate objective indicators of biological maturation to better distinguish developmental changes from sport-specific adaptations and to establish reference values across different stages of adolescence [1,2,6].

Despite its limitations, the present study possesses several important strengths. To the authors’ knowledge, relatively few investigations have compared body composition characteristics across individual, team, and combat sports within the same cohort of adolescent athletes enrolled in a structured sports technification program. Furthermore, all anthropometric measurements were performed by the same ISAK-accredited anthropometrist using standardized procedures, thereby minimizing measurement variability and enhancing methodological consistency. The simultaneous application of three widely used prediction equations also provides complementary information regarding adiposity assessment and increases the value of the present findings for future comparisons involving young athletic populations [12,16].

Taken together, the present findings support the view that body composition in adolescent athletes should be interpreted within the specific context of each sporting discipline rather than according to universal normative standards. The interaction between biological growth, maturation, and sport-specific training stimuli appears to contribute to the development of distinct anthropometric profiles that may influence both athletic performance and long-term athlete development [6]. Consequently, standardized anthropometric assessment performed according to internationally accepted protocols may represent a valuable tool for monitoring morphological adaptations, guiding individualized training and nutritional strategies, and facilitating evidence-based decision-making within sports technification programs [12].

Although the present investigation included a comprehensive anthropometric assessment following the ISAK protocol, the current manuscript specifically focuses on body composition comparisons among sport categories. A more detailed characterization of the same cohort, including skinfold thicknesses, girths, bone breadths, proportionality indices, and somatotype analysis, has been reported elsewhere by the same research group. Consequently, these variables are not reproduced in the present manuscript in order to avoid duplication and to maintain the specific scope of this study [23].

Importantly, the present study addresses a distinct analytical question by specifically comparing estimated body composition components across the predefined sport categories and examining whether the observed adiposity patterns are consistent across three different anthropometric prediction equations. These comparative analyses and the corresponding between-category statistical contrasts were not the focus of the previous publication. Therefore, rather than providing a second general anthropometric description of the cohort, the present study extends the previous work by examining body composition as a specific outcome and by evaluating differences across sport categories using complementary estimation approaches.

### Limitations

The present study has several limitations that should be acknowledged. First, its cross-sectional design precludes establishing causal relationships between participation in a specific sport category and the body composition characteristics observed. Longitudinal investigations are therefore warranted to determine how these anthropometric profiles evolve throughout adolescence and in response to long-term training exposure.

Second, biological maturation and pubertal status were not directly assessed and may have acted as potential confounding factors influencing the differences identified among participants, given the considerable physiological changes that occur during this developmental period [6]. Furthermore, male and female athletes were included together in the primary comparisons between sport categories. Given the well-established sex-related differences in body composition during adolescence, particularly in the context of biological maturation, residual confounding by sex cannot be excluded, even though the distribution of male and female participants was relatively balanced across the three predefined sport categories. The limited sample sizes within individual sport- and sex-specific subgroups precluded sufficiently powered stratified inferential analyses. Therefore, the present findings should be interpreted as exploratory overall comparisons between the specific groups of athletes included in the study rather than as sex-specific body composition differences. Future studies with larger samples should incorporate sex-stratified analyses and objective indicators of biological maturation to better account for these potential sources of variability.

Third, body composition was estimated using anthropometric prediction equations rather than criterion methods such as dual-energy X-ray absorptiometry (DXA). Although all measurements were obtained according to standardized ISAK procedures by an accredited anthropometrist, with acceptable intra-rater measurement reliability [12,16], indirect estimation methods may still produce values that differ from those obtained using imaging techniques.

An additional limitation concerns the exploratory body-size-adjusted sensitivity analysis. Although body-size-adjusted anthropometric variables (ISAK Phantom Z-scores) together with fat mass index (FMI) and fat-free mass index (FFMI) were examined to explore the potential influence of body size, individual ISAK assessment files were available only for athletes participating in handball, volleyball, karate, and taekwondo. Consequently, this supplementary analysis could not incorporate the athletics, swimming, and triathlon groups included in the primary study. Therefore, the body-size-adjusted analyses should be interpreted as exploratory and should not be considered a direct size-adjusted replication of the primary comparisons among the predefined individual, team, and combat sport categories.

Finally, the relatively small sample sizes within some sport-specific subgroups may have limited the statistical power to detect subtle between-group differences and restricted the possibility of conducting robust subgroup analyses. Although adjustment for multiple pairwise comparisons reduced the risk of Type I error, the relatively small sample size and the number of outcomes examined should still be considered when interpreting the statistical findings.

## 5. Conclusions

The present exploratory study identified differences in selected body composition indicators among young athletes participating in the specific individual (athletics, swimming, and triathlon), team (handball and volleyball), and combat (karate and taekwondo) sports included in this Spanish Sports Technification Program. Within this cohort, athletes from the included individual sports generally exhibited lower body fat percentages than those from the included team and combat sports, whereas differences were also observed in the distribution of muscle and bone mass across the analyzed sport categories. These findings should be interpreted specifically within the context of the sports and athletes included in the present study and should not be generalized to individual, team, or combat sports as a whole.

The observed differences may reflect, at least in part, the distinct physiological and performance demands of the sports represented in this cohort, although the potential influence of sex, biological maturation, playing position, and competitive weight category should also be considered. Accordingly, standardized body composition assessment may be useful for monitoring athlete development and informing training and nutritional decision-making when interpreted within the specific context of each sport and athlete.

In practical terms, the descriptive and comparative data reported here may help coaches, sports scientists, nutritionists, physiotherapists, and other health professionals to interpret body composition in youth athletes participating in similar sports and development programs. Future studies should use longitudinal designs and include biological maturation and training load as well as sex- and sport-specific factors to better clarify how sport participation influences body composition over time.

Beyond the descriptive findings, the present study provides standardized comparative reference data obtained under homogeneous assessment conditions, which may facilitate the contextual interpretation of body composition in young athletes participating in similar sports development programs.

## Figures and Tables

**Table 1:** Demographic and anthropometric characteristics of the study participants according to sport category.

Variable	Category	Individual Sports	Team Sports	Combat Sports
Participants (*n*)	Female	15	21	20
	Male	23	20	19
	Total	38	41	39
Age (years)	Female	14.91 (1.39)	15.23 (0.80)	14.87 (1.17)
	Male	15.30 (1.34)	14.84 (0.88)	14.66 (1.48)
	Total	15.16 (1.35)	15.04 (0.85)	14.76 (1.32)
Body mass (kg)	Female	53.53 (6.00)	59.76 (7.74)	53.95 (8.86)
	Male	59.78 (10.07)	68.03 (12.34)	55.75 (14.05)
	Total	57.51 (9.24)	63.89 (11.00)	54.83 (11.57)
Stature (cm)	Female	161.36 (5.13)	165.40 (4.66)	161.12 (7.90)
	Male	170.68 (9.72)	175.94 (11.24)	163.69 (11.62)
	Total	167.29 (9.44)	170.77 (10.03)	162.37 (9.85)
Humerus breadth (cm)	Female	5.65 (0.29)	6.10 (0.57)	5.51 (0.47)
	Male	6.46 (0.51)	6.89 (0.55)	6.10 (0.89)
	Total	6.14 (0.59)	6.49 (0.68)	5.80 (0.76)
Femur breadth (cm)	Female	8.87 (0.50)	9.44 (0.56)	8.95 (0.72)
	Male	10.10 (1.50)	10.11 (0.95)	9.76 (1.14)
	Total	9.62 (1.34)	9.77 (0.84)	9.29 (1.00)

Data are presented as mean (standard deviation) unless otherwise indicated.

**Table 2:** Body composition characteristics and pairwise comparisons among athletes participating in individual, team, and combat sports.

Variable	Individual Mean ± SD	Team Mean ± SD	Combat Mean ± SD	Individual vs. Team Adj. *p*	r (95% CI)	Individual vs. Combat Adj. *p*	r (95% CI)	Team vs. Combat Adj. *p*	r (95% CI)
Fat mass (%)	29.68 ± 1.03	30.03 ± 1.30	30.20 ± 0.89	NR	NR	NR	NR	NR	NR
Fat mass (kg)	17.18 ± 2.97	19.29 ± 3.72	16.76 ± 3.72	0.056	−0.26 (−0.45, −0.04)	NR	NR	**0.004**	−0.36 (−0.54, −0.15)
Bone mass (%)	3.92 ± 1.09	3.44 ± 0.76	3.85 ± 1.05	0.088	−0.25 (−0.45, −0.03)	NR	NR	NR	NR
Bone mass (kg)	2.20 ± 0.45	2.15 ± 0.28	2.05 ± 0.32	NR	NR	NR	NR	NR	NR
Muscle mass (%)	36.28 ± 3.20	34.02 ± 3.21	35.68 ± 3.56	**0.023**	−0.30 (−0.49, −0.08)	NR	NR	0.110	−0.23 (−0.43, −0.01)
Muscle mass (kg)	20.89 ± 3.46	21.63 ± 3.24	19.46 ± 2.72	NR	NR	NR	NR	**0.007**	−0.34 (−0.52, −0.13)
Withers body fat (%)	15.17 ± 2.06	18.25 ± 3.69	16.93 ± 2.64	**<0.001**	−0.44 (−0.60, −0.24)	**0.006**	−0.35 (−0.53, −0.14)	NR	NR
Faulkner body fat (%)	13.55 ± 4.32	17.49 ± 6.22	15.85 ± 4.74	**0.007**	−0.34 (−0.52, −0.13)	**0.038**	−0.28 (−0.47, −0.06)	NR	NR
Carter body fat (%)	11.05 ± 4.44	15.34 ± 6.65	13.46 ± 4.82	**0.004**	−0.36 (−0.54, −0.15)	**0.026**	−0.30 (−0.49, −0.08)	NR	NR

Data are presented as mean ± standard deviation. Pairwise comparisons were performed using the Mann–Whitney U test following the overall Kruskal–Wallis analysis, when appropriate. Adjusted *p*-values were obtained using Bonferroni correction for the three possible pairwise comparisons for each outcome. Effect sizes (r) were calculated from the standardized Z statistic. Approximate 95% confidence intervals (CI) for effect sizes were estimated using Fisher’s z transformation and were not adjusted for multiple comparisons. NR, not reported because the corresponding pairwise test statistic was not available from the original statistical output. Bold values indicate statistical significance after Bonferroni correction (adjusted *p* < 0.05).

## Data Availability

The data presented in this study are available from the corresponding author upon reasonable request. The data are not publicly available due to privacy and ethical considerations related to the participants.

## Supplementary Materials

The following supporting information can be downloaded at: https://www.mdpi.com/article/10.3390/jfmk11030299/s1.

## Abbreviations

The following abbreviations are used in this manuscript:

ISAK	International Society for the Advancement of Kinanthropometry
IBM	International Business Machines
SPSS	Statistical Package for the Social Sciences
DXA	Dual-Energy X-ray Absorptiometry
Mdn	Median
