# Supplementary material for: Body Composition Differences Among Individual, Team, and Combat Sport Athletes Enrolled in a Spanish Sports Technification Program
Source: J Funct Morphol Kinesiol. 2026 Jul 29;11(3):299. doi: 10.3390/jfmk11030299 (PMC13510554; doi:10.3390/jfmk11030299)
Supplement: Supplementary file 1 [file jfmk-11-00299-s001.zip › jfmk-4410031-supplementary.pdf]

Supplementary Table S1. Body composition characteristics of athletes participating in individual sports, stratified by sport discipline and sex.

Measure	Athletics Female (n = 6)	Athletics Male (n = 6)	Swimming Female (n = 5)	Swimming Male (n = 8)	Triathlon Female (n = 4)	Triathlon Male (n = 9)
Fat mass (%)	29.79 ± 0.68	29.02 ± 1.21	30.83 ± 1.12	29.18 ± 0.47	30.20 ± 0.78	29.60 ± 1.05
Fat mass (kg)	15.25 ± 2.04	16.33 ± 4.09	17.59 ± 1.26	17.53 ± 1.60	15.47 ± 2.39	19.24 ± 3.59
Bone mass (%)	3.95 ± 0.65	4.76 ± 1.77	3.27 ± 0.36	3.97 ± 0.54	3.85 ± 0.80	3.67 ± 1.35
Bone mass (kg)	1.99 ± 0.15	2.47 ± 0.20	1.86 ± 0.14	2.36 ± 0.22	1.93 ± 0.15	2.33 ± 0.77
Muscle mass (%)	34.38 ± 2.67	35.56 ± 3.46	36.03 ± 2.75	37.59 ± 2.90	37.03 ± 4.51	36.67 ± 3.35
Muscle mass (kg)	17.46 ± 1.19	19.74 ± 4.05	20.52 ± 1.52	22.45 ± 0.95	18.71 ± 1.10	23.75 ± 4.22
Withers body fat (%)	16.30 ± 0.94	13.34 ± 0.96	17.11 ± 2.78	14.35 ± 1.10	17.62 ± 1.98	14.21 ± 1.26
Faulkner body fat (%)	16.83 ± 1.94	9.83 ± 0.41	18.60 ± 4.39	11.50 ± 3.21	18.25 ± 3.30	10.78 ± 1.72
Carter body fat (%)	14.50 ± 1.52	7.17 ± 0.75	16.40 ± 4.51	8.75 ± 2.66	16.25 ± 3.30	8.11 ± 1.54

Note: Data are presented as mean ± standard deviation.

Supplementary Table S2. Body composition characteristics of athletes participating in team sports, stratified by sport discipline and sex.

Measure	Handball Female (n = 6)	Handball Male (n = 11)	Volleyball Female (n = 15)	Volleyball Male (n = 9)
Fat mass (%)	30.63 ± 1.32	29.53 ± 1.58	30.29 ± 1.00	29.79 ± 1.30
Fat mass (kg)	19.36 ± 3.15	21.16 ± 4.86	17.77 ± 2.76	19.48 ± 3.29
Bone mass (%)	3.14 ± 0.76	3.27 ± 0.67	3.60 ± 0.72	3.58 ± 0.95
Bone mass (kg)	1.94 ± 0.29	2.27 ± 0.28	2.07 ± 0.26	2.26 ± 0.24
Muscle mass (%)	32.32 ± 2.49	35.32 ± 3.06	34.31 ± 3.57	33.09 ± 2.81
Muscle mass (kg)	20.27 ± 2.27	24.96 ± 3.77	19.84 ± 0.88	21.46 ± 2.74
Withers body fat (%)	21.15 ± 1.78	15.29 ± 2.36	20.61 ± 3.61	16.00 ± 1.31
Faulkner body fat (%)	22.50 ± 3.56	12.64 ± 3.53	22.00 ± 5.22	12.56 ± 2.40
Carter body fat (%)	21.17 ± 3.25	9.64 ± 2.87	20.40 ± 5.40	10.00 ± 1.66

Note: Data are presented as mean ± standard deviation.

Supplementary Table S3. Body composition characteristics of athletes participating in combat sports, stratified by sport discipline and sex.

Measure	Karate Female (n = 7)	Karate Male (n = 13)	Taekwondo Female (n = 13)	Taekwondo Male (n = 6)
Fat mass (%)	30.63 ± 0.98	30.03 ± 1.13	30.15 ± 0.56	30.19 ± 0.87
Fat mass (kg)	18.14 ± 3.55	16.33 ± 4.03	15.61 ± 1.96	18.60 ± 5.60
Bone mass (%)	3.05 ± 0.47	4.21 ± 0.91	3.83 ± 0.73	4.06 ± 1.90
Bone mass (kg)	1.77 ± 0.16	2.21 ± 0.36	1.95 ± 0.24	2.23 ± 0.23
Muscle mass (%)	35.57 ± 3.63	36.92 ± 2.94	35.50 ± 2.73	33.53 ± 5.69
Muscle mass (kg)	20.75 ± 1.99	19.76 ± 3.21	18.26 ± 1.62	19.89 ± 3.75
Withers body fat (%)	19.93 ± 2.45	15.99 ± 2.07	16.76 ± 1.75	15.83 ± 3.43
Faulkner body fat (%)	21.43 ± 4.83	12.69 ± 3.04	17.23 ± 3.06	13.17 ± 3.87
Carter body fat (%)	19.71 ± 4.46	10.23 ± 2.80	14.85 ± 2.85	10.17 ± 3.82

Note: Data are presented as mean ± standard deviation.

Supplementary Table S4. Exploratory comparison of body-size-adjusted anthropometric and body composition variables among handball, volleyball, karate, and taekwondo athletes (n = 80).

Variable	Kruskal–Wallis H	p-value	ε^2	Significant pairwise comparisons (Bonferroni-adjusted)
BMI	4.71	0.194	0.022	None
Fat Mass Index (FMI)	3.25	0.354	0.003	None
Fat-Free Mass Index (FFMI)	5.47	0.140	0.032	None
Weight Phantom Z-score	0.47	0.926	0.000	None
Triceps Phantom Z-score	1.84	0.607	0.000	None
Subscapular Phantom Z-score	7.17	0.067	0.055	None
Biceps Phantom Z-score	1.50	0.683	0.000	None
Iliac Crest Phantom Z-score	2.06	0.560	0.000	None
Supraspinale Phantom Z-score	2.14	0.543	0.000	None
Abdominal Phantom Z-score	NS	>0.05	–	None
Front Thigh Phantom Z-score	NS	>0.05	–	None
**Medial Calf Phantom Z-score**	**10.80**	**0.013**	**0.103**	**Volleyball vs Taekwondo (adjusted p = 0.016)**
Arm Girth Phantom Z-score	NS	>0.05	–	None
Waist Girth Phantom Z-score	NS	>0.05	–	None
Hip Girth Phantom Z-score	NS	>0.05	–	None
**Calf Girth Phantom Z-score**	**9.62**	**0.022**	**0.087**	**Volleyball vs Taekwondo (adjusted p = 0.044); Karate vs Taekwondo (adjusted p = 0.034)**
**Humerus Breadth Phantom Z-score**	**9.26**	**0.026**	**0.082**	No pairwise comparison remained significant after Bonferroni correction
Femur Breadth Phantom Z-score	NS	>0.05	–	None

Abbreviations: *BMI, body mass index; FMI, fat mass index; FFMI, fat-free mass index; ε^2, epsilon-squared effect size; NS, not statistically significant ($p > 0.05$).*

Footnote: *Body-size-adjusted analyses were performed as an exploratory sensitivity analysis using the available individual ISAK assessment files from handball, volleyball, karate, and taekwondo athletes. Two incomplete records were excluded, resulting in a final sample of 80 athletes (17 handball, 24 volleyball, 20 karate, and 19 taekwondo). Overall differences were assessed using the Kruskal–Wallis test. Pairwise comparisons were performed using the Mann–Whitney U test with Bonferroni adjustment when appropriate.*
